# Disrupting pathologic phase transitions in neurodegeneration

**DOI:** 10.1172/JCI168549

**Published:** 2023-07-03

**Authors:** Bryan T. Hurtle, Longxin Xie, Christopher J. Donnelly

**Affiliations:** 1 Center for Neuroscience at the University of Pittsburgh Graduate Program;; 2 Medical Scientist Training Program, University of Pittsburgh; and; 3 LiveLikeLou Center for ALS Research at the University of Pittsburgh Brain Institute; Pittsburgh, Pennsylvania, USA.; 4 Department of Neurobiology, University of Pittsburgh School of Medicine, Pittsburgh, Pennsylvania, USA.; 5 School of Medicine, Tsinghua University, Beijing, China.

## Abstract

Solid-like protein deposits found in aged and diseased human brains have revealed a relationship between insoluble protein accumulations and the resulting deficits in neurologic function. Clinically diverse neurodegenerative diseases, including Alzheimer’s disease, Parkinson’s disease, frontotemporal lobar degeneration, and amyotrophic lateral sclerosis, exhibit unique and disease-specific biochemical protein signatures and abnormal protein depositions that often correlate with disease pathogenesis. Recent evidence indicates that many pathologic proteins assemble into liquid-like protein phases through the highly coordinated process of liquid-liquid phase separation. Over the last decade, biomolecular phase transitions have emerged as a fundamental mechanism of cellular organization. Liquid-like condensates organize functionally related biomolecules within the cell, and many neuropathology-associated proteins reside within these dynamic structures. Thus, examining biomolecular phase transitions enhances our understanding of the molecular mechanisms mediating toxicity across diverse neurodegenerative diseases. This Review explores the known mechanisms contributing to aberrant protein phase transitions in neurodegenerative diseases, focusing on tau and TDP-43 proteinopathies and outlining potential therapeutic strategies to regulate these pathologic events.

## Introduction

Neurodegenerative diseases (NDDs) are a heterogeneous class of incurable and debilitating disorders characterized by the progressive degeneration of vulnerable cell populations in the central nervous system (CNS). Decades of research investigating the most common NDDs, such as Alzheimer’s disease (AD), Parkinson’s disease (PD), frontotemporal lobar degeneration (FTLD), and amyotrophic lateral sclerosis (ALS), revealed clinical and neuropathologic heterogeneity between, and within, these diseases (1–3). However, NDDs display a fundamental commonality — proteins soluble under physiologic conditions accumulate into solid-like pathologic protein inclusions, and this is associated with clinical progression (4, 5). Furthermore, disease-causing mutations in genes that encode proteins that pathologically accumulate, such as amyloid-β (*APP*) in AD, tau (*MAPT*) in FTLD-tau, TDP-43 (*TARDBP*) in ALS/FTLD–TDP-43, and α-synuclein (*SNCA*) in PD, cause familial forms of each disease (6–8). Sporadic-NDD patients with unclear familial inheritance and no genetic mutation in the genes that encode these proteins similarly present with neuropathologic deposits of the wild-type protein in the CNS. Furthermore, these sporadic NDDs often display remarkably similar clinical syndromes when compared with the familial form of the disease (2, 9).

Multidisciplinary efforts have gone into understanding mechanisms through which tau and TDP-43 proteins regulate neuronal homeostasis and contribute to NDDs (10–13). These efforts revealed considerable clinical overlap between tau and TDP-43 proteinopathies (14–16). In addition to AD, the most common NDD, solid self-assemblies of tau are found in related dementias termed “tauopathies,” including FTLD-tau, corticobasal degeneration (CBD), Pick’s disease, progressive supranuclear palsy (PSP), and chronic traumatic encephalopathy (CTE) (17). While several mutations in the *TARDBP* gene contribute to a small percentage of ALS and FTLD–TDP-43 cases, mislocalized and insoluble TDP-43 self-assemblies are found in up to about 97% of individuals with sporadic ALS, up to about 85% with CTE, about 45% with FTLD, and about 40%–60% with AD (18, 19). Several recent studies characterized the ability of these proteins to undergo liquid-like phase separation under physiologic conditions, often into membraneless organelles (13, 20–24). Accordingly, the incidence of tau and TDP-43 pathology across genetic and sporadic NDDs likely highlights a convergence of several upstream mechanisms driving aberrant protein phase transitions and disease progression.

In the following sections, we will explore the relationship between protein structure, biological phase transitions, protein self-assembly, and the organization of multicomponent condensates using tau and TDP-43 as representative proteins. Later sections will survey diverse targeting strategies proposed for tau and TDP-43 proteinopathies, focusing on how protein phase transitions and condensate assembly mechanisms can be leveraged as potential therapeutic avenues of intervention.

## Protein self-assembly through homotypic phase transitions

Intracellular NDD-associated proteins self-assemble into diverse polymeric structures and liquid-like protein phases capable of organizing functionally related proteins, nucleic acids, and various biomolecules (13, 22, 25–27). Physiologically, most proteins are soluble and exist in a liquid-like state. In a simple system, e.g., a purified protein in solution, a protein will be soluble when the attractive interactions between different molecules are low enough to maintain a well-mixed state (26, 28, 29). Raising the protein concentration or modifying the balance of attractive and repulsive forces may exceed a protein’s saturation concentration (*C*_sat_) and precipitate a new and denser phase. In this context, where the protein is lacking complex biomolecular interactions, homotypic interactions regulate a segregative protein phase transition, which results in a new, denser phase (*C*_dense_) coexisting within the dilute phase (28, 30, 31). Strong driving forces for a given phase transition are generated by lowering of the *C*_sat_, a context-dependent property affected by factors intrinsic to a protein’s sequence, localized concentration, cell size changes, temperature, pH, and ionic environments (28, 32–35).

Phase transitions that give rise to two coexisting phases can be liquid-like or display solid-like properties. The appropriate prefix (liquid-like, solid-like) depends on the material properties of the emerging phase. Defining characteristics that distinguish liquid-like protein phases include rapid reversibility, interior molecular diffusion, and the ability to exchange molecules with the surrounding phase (i.e., cytosol) (26, 36). Notably, liquids may transition into solid-like states that emerge through several processes, including gelation, requiring networks of interactions (gel-like), or age-dependent increases in viscosity (glass-like). Solids can also emerge from liquid-like phases by forming fibrils or crystal-like aggregates such as amyloids.

The human proteome is a continuum of protein structures ranging from intrinsically folded proteins to intrinsically disordered proteins (IDPs), with most containing both ordered domains and intrinsically disordered regions (IDRs) (28, 37–39). IDRs are regions of a protein sequence that lack well-defined secondary and tertiary structures. Phase separation and condensate-promoting features include modular interaction domains and stretches of low-complexity sequences found within IDRs. Tau and TDP-43 are modular, multivalent proteins with IDRs that enable and regulate homotypic and heterotypic interactions to generate complex and context-dependent molecular interactions (10, 23, 40–42). These protein architectures tune the concentration required for phase separation and dictate the resultant assembly and material states. Importantly, proteins with IDRs exist in a dynamic equilibrium of conformationally distinct states, and the structural properties of IDRs can quickly adjust owing to changes in solution conditions, posttranslational modifications (PTMs), or interactions with other molecules (26, 34, 36, 43–45).

The generation of neurotoxic self-assemblies represents a fundamental transformation during the pathogenesis of NDDs (4, 5, 17, 46–48). In vitro experiments using purified proteins, work in transgenic animal disease models, and studies with postmortem human brain tissue show that both tau and TDP-43 self-assemble into polymeric states with varying structures and material properties (17, 49–59). Self-polymerization can occur under dilute conditions or within liquid-like droplets. However, pathogenic tau and TDP-43 self-assemblies rely on the exposure of small aggregation-prone regions, including steric zippers or low-complexity, aromatic-rich, kinked segments (LARKS) (60, 61). Disruption in protein conformation due to intrinsic factors (NDD-causing missense mutations, PTMs) or extrinsic factors (biomolecular interactions, cellular environment) may expose these buried short, aggregation-prone sequences in proteins (24, 62). Importantly, specific conformational transformations of protein monomers that are capable of nucleating stable self-interactions with other monomers are required (48, 63). These structural conformations drive unique assembly pathways specific to that protein, which ultimately translates to distinct pathologies observed in NDDs (64). Recent groundbreaking cryo–electron microscopy studies found that specific conformations underlie clinical subtypes of tau and TDP-43 pathologies. Remarkably, an increasing number of clinical presentations and neuropathologic findings correlate with structurally specific fibrils of varying biophysical properties and cellular effects (51–53, 56–58).

**Figure 1:**
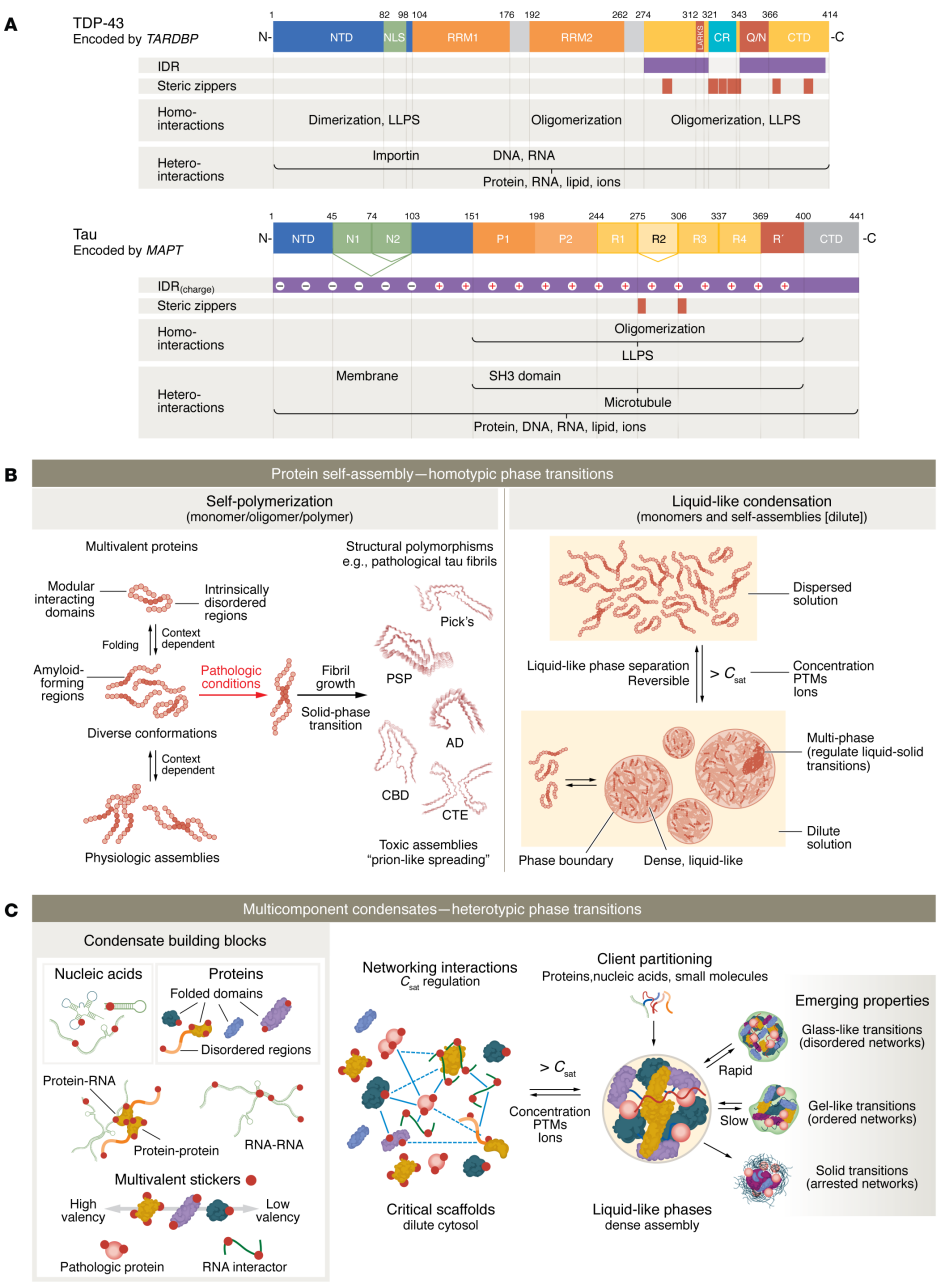
Phase transitions of NDD-related proteins. (**A**) Domain structure and interaction features of TDP-43 and tau. TDP-43 contains three domains: an N-terminal domain (NTD) including a nuclear localization sequence (NLS); two RNA recognition motifs (RRM1 and RRM2); and a C-terminal domain (CTD) with a short α-helical fold (CR helix) and a glutamine/arginine-rich region (Q/N). Tau contains four domains: the negatively charged NTD and CTD, and the positively charged proline-rich domain (P1–P2) and microtubule-binding domain (MTBD; R1–R4). Six different tau isoforms are generated by alternative splicing containing zero, one, or two NTD inserts and three or four MTBD repeats. The intrinsically disordered regions (IDRs), aggregation-prone steric zippers, and domain-dependent homo/heterotypic biomolecular interactions of TDP-43 and tau are shown accordingly. LLPS, liquid-liquid phase separation. (**B**) Aberrant protein conformations, toxic polymeric self-assemblies, and solid accumulations of proteins are found across the most common NDDs. TDP-43 and tau are modular, multivalent proteins exhibiting conformational flexibility, allowing diverse monomeric conformations, polymeric assemblies, and liquid-like phase behaviors in normal physiology and pathology. Sequence-specific properties found within distinct protein domains (modular interaction domains, intrinsically disordered regions, and amyloid-forming regions) are influenced by intrinsic (isoforms, mutations, PTMs) and extrinsic factors (molecular interactions, environmental conditions), ultimately regulating phase behavior and unique polymerization pathways. While increased homotypic interactions drive protein self-polymerization and the phase separation of proteins into liquid-like droplets, they are independent processes regulated by overlapping conditions. (**C**) TDP-43 and tau reside within multicomponent biomolecular condensates and thus are subjected to diverse homo/heterotypic biomolecular interactions, ultimately regulating physiologic and pathologic phase transitions. Biomolecules necessary for condensate assembly (scaffolds) spatially organize and concentrate functionally related biomolecules (clients) through liquid-like phase transitions. A sticker and spacer model has been proposed in which sticker sequences regulate multivalent networking interactions and spacer sequences regulate the solubilities of individual biomolecules and emerging networks.

While short aggregation-prone sequences necessary for pathologic self-assemblies have been discovered in tau and TDP-43 (steric zippers and LARKS), several other regions within these two proteins can regulate phase transition behavior. TDP-43 contains a C-terminal domain (CTD) that comprises two disordered regions (IDR1, IDR2) and a short α-helical fold (CR helix) that is stabilized by adjacent homomeric contacts between other TDP-43 CR helices (Figure 1A) (23, 65). This CTD, also defined as a low-complexity domain (LCD), is sufficient for liquid-like phase separation and likely important for physiologic functions, including RNA splicing (24, 66, 67). However, current experimental evidence supports a TDP-43 oligomerization model where physically distant regions regulate the ability to nucleate self-assembly. While the N-terminal domain (NTD) drives the physiologic self-assembly necessary for RNA binding through two canonical RNA recognition motifs, the C-terminal LCD, a region where most ALS/FTLD-associated mutations are found, mediates dysfunctional assemblies (24, 68–71). Segments of the TDP-43 LCD can form both steric zippers and LARKS. Interestingly, the NTD appears to resist self-assemblies regulated by pathologic (LCD) self-interactions (72). Similarly, tau protein can be divided into distinct motifs: the negatively charged NTD and CTD and the positively charged proline-rich domain (PRD) and microtubule-binding domain (MTBD) (Figure 1B). Six different tau isoforms are generated in the human brain by alternative splicing containing varying NTD inserts (zero, one, or two) and three or four MTBD repeats. Importantly, tau contains two aggregation-prone steric zipper motifs within the MTBD (10), and while both the PRD and MTBD domains are capable of phase separation, the PRD has a prominent role in regulating tau liquid-liquid phase separation in cells (42).

## Condensate assembly through heterotypic phase transitions

Membrane-bound structures were historically considered the established systems of intracellular organization. However, emerging research has since highlighted the role of dynamic biomolecular condensates, commonly referred to as membraneless organelles, as another process underlying cellular compartmentalization. These are dynamic assemblies formed through phase transitions consisting of homotypic/heterotypic interactions between proteins, nucleic acids, and cofactors (26, 28, 36). Hundreds or thousands of intracellular biomolecular interactions (“heterotypic buffering effect”) that occur under physiologic conditions prevent deleterious homotypic protein interactions observed in NDDs (22, 28). Thus, a better understanding of liquid-like phases and their liquid-to-solid transitions is important for understanding NDD pathogenesis.

A leading hypothesis for condensate assembly defines the condensate components as either scaffolds or clients. Scaffolds ultimately regulate the incorporation of various client biomolecules, which are not necessary for condensate assembly but essential for condensate dynamics and function. Subsequently, necessary scaffold-client interactions drive the assembly and tune the dynamic compositions of biomolecular condensates (33, 43, 73, 74). RNA species are integral components of many described condensates and, like proteins, are capable of scaffolding condensates through multivalent interactions (24, 75–83). Furthermore, RNA can promote or dissolve condensates scaffolded by RNA-binding proteins (RBPs), likely depending on their sequence, structure, and valence (23, 75, 83, 84).

Scaffolding molecules encode structural elements that drive and regulate phase transitions, including the generation of pre-assemblies such as small clusters and/or liquid-like phases. Multivalence is a common feature of scaffolding molecules and acts as a critical regulator of heterotypic phase transitions (35, 39, 73, 74, 79). Multivalence can be achieved in several ways, though it generally involves weak, transient contacts through modular interaction domains. Scaffolding protein motifs participating in specific interactions can occur on folded domains, low-complexity motifs, and sometimes even single residues. These interaction motifs form reversible cross-links through various chemical interactions, referred to as stickers (73, 74, 85). Sticker motifs are the same cross-links that drive protein folding, fold-specific recognition motifs, and many “classic” molecular assemblies known in biology (74). Additionally, while stickers engage in physical cross-links, various spacer sequences within the protein impact its overall solubility.

Biomolecular condensation can be coupled to both segregative phase separation (density transitions) and percolation, an associative phase transition (or networking transitions), as well as cooperative density-driven network transitions (phase separation coupled to percolation) (28). In a percolated network, physically cross-linked networks form via liquid-to-gel transitions, leading to network-spanning structures (28). The valence of stickers and their interaction strengths define the intrinsic concentration, or “percolation threshold” (*C*_perc_), necessary for networking phase transitions (28, 74). Client-scaffold binding significantly alters saturation concentrations required for assembly and dissolution, providing switch-like, rapid behavior. Therefore, a networking transition is enabled by specific interactions between biomolecules (scaffolds) with a multivalence of interaction motifs (stickers). Regardless of the mechanisms driving their assembly, the ability to locally concentrate specific biomolecules is a classic description of all discovered biomolecular condensate structures (Figure 1C).

## Cellular functions of biomolecular condensates

**Figure 2:**
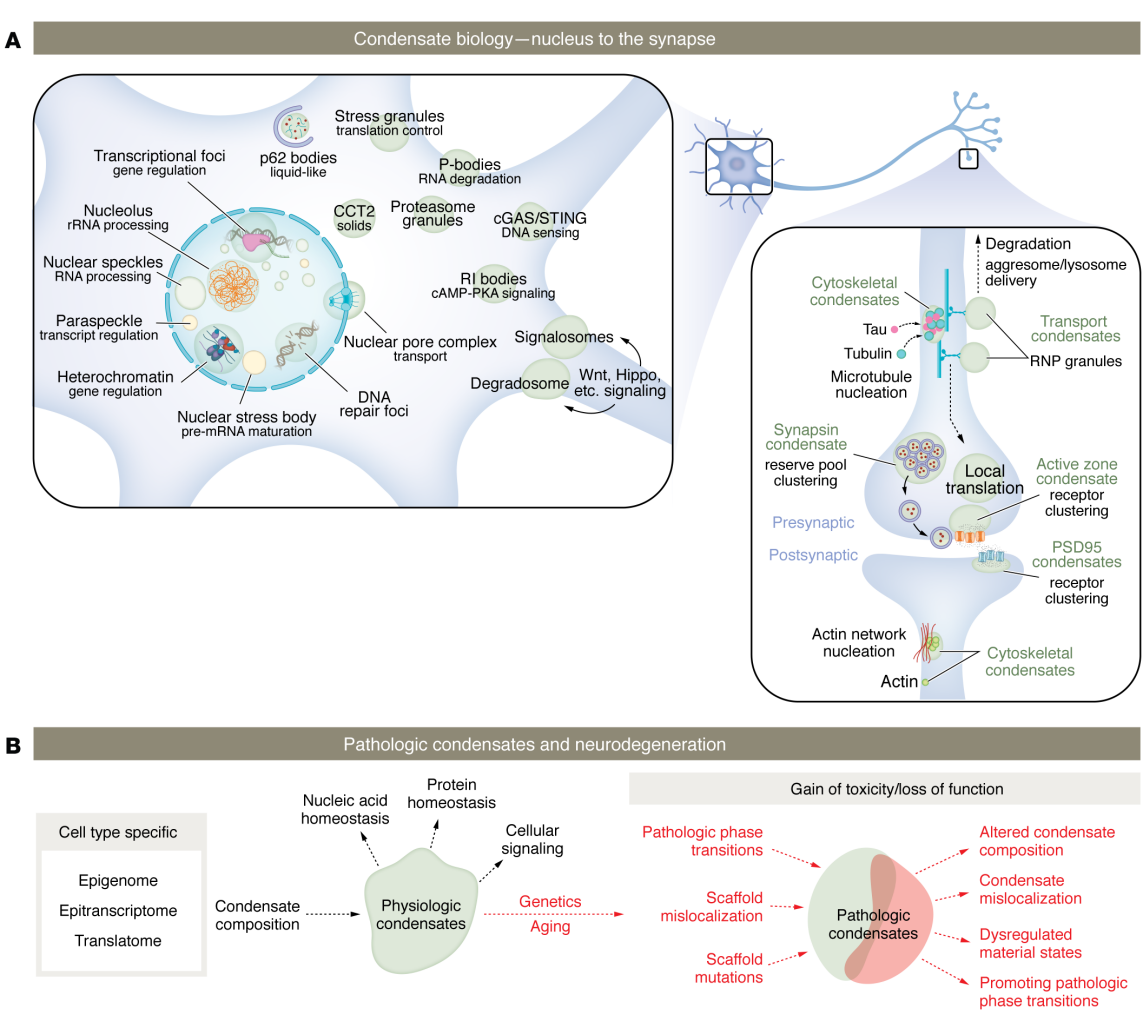
Hallmarks of neurodegeneration involve functions related to diverse biomolecular condensates. (**A**) Schematic diagram showing the localization of various biomolecular condensates in a neuronal cell. Various biomolecular condensates are associated with many cellular processes that influence the homeostasis of nucleic acids and proteins from the nucleus to the end of synapses. (**B**) Neurodegeneration is accompanied by genetic, transcriptomic, and translational disruptions within vulnerable, cell type–specific neuronal populations. Imbalances in nucleic acid and protein homeostasis will directly affect the compositions, localization, and function of condensates (loss of function), additionally leading to aberrant phase transitions occurring within pathologic condensates (gain of function). Additionally, pathologic protein assemblies lead to downstream disruptions of physiologic condensates. RI, type I regulatory subunity of cAMP-dependent protein kinase (PKA).

Today, biomolecular condensates are thought to spatially organize related processes in compartments ranging from the nucleus to the end of neuronal synapses (Figure 2A) (86–88). Primarily, condensates act as organization hubs, allowing spatiotemporal control of a variety of localized functions. They can also act as reaction crucibles, where the concentration of molecules in a condensed state promotes dynamic exchanges of products/reactants and sequesters biomolecules for storage or degradation. By spatiotemporally organizing biomolecules, unique biomolecular condensates dictate the biosynthesis, transport, regulation, and function of the basic building blocks necessary for cellular homeostasis (28, 30, 32, 36, 89, 90).

In the absence of membrane-bound elements, distinct condensates can regulate nuclear functions, including chromatin compaction, DNA repair, RNA transcription, processing, transport, and decay (29, 89, 91–95). The most widely known subnuclear biomolecular condensate is the nucleolus. Nucleoli are multiphase condensates present within all eukaryotic organisms and are known as the site of rRNA transcription and ribosome assembly. The liquid-like state of nucleoli allows for a rapid exchange of newly transcribed/processed rRNA and ribosomal subunits between subcompartments of the nucleolus, permitting proper assembly and export of ribosomes from the nucleus (89, 94, 96–98). Phase-separated condensates are also implicated in driving gene activation through transcriptional condensates assembled at enhancer-rich gene clusters (95, 99–101). Properties inherent to chromatin, including the spacing of nucleosomes, allow it to phase-separate within the nucleoplasm, thus enabling the establishment and maintenance of distinct chromatin subcompartments (91, 102). Other well-studied nuclear condensates worth mentioning include Cajal bodies (associated with maturation of spliceosomal RNA and small nuclear ribonucleoprotein complexes), paraspeckles (involved in RNA editing and a protein buffering reservoir), nuclear speckles (“assembly line” involved in transcription-splicing mRNA export), and promyelocytic leukemia (PML) bodies (implicated in DNA damage and telomere maintenance) (89, 92).

Another well-known biomolecular condensate is cytoplasmic stress granules (SGs). SGs are a considerable focus in the field of neurodegeneration following the discovery that several disease-linked RBPs, including TDP-43 and tau, can localize to and modify SG assembly and dynamics (103). This micrometer-sized condensate assembles RNA and RBPs under various cellular stressors, and these structures regulate RNA stability and triage non-essential protein translation until the stress is removed (103–109). Recent studies show that the initial pre-assembly of G3BP1/2 dimers (which promote liquid-liquid phase separation) and the newly released mRNAs from polysomes during translational inhibition provide a physical platform for SG assembly. This initial assembly process is then followed by the subsequent recruitment of client molecules required for SG condensation and function necessary during cellular stress (79, 109).

During the last several years, studies demonstrate the importance of liquid-like condensation with regard to the spatiotemporal organization of neurons (86–88, 110). This growing group of structures includes synaptic active zones, synaptic vesicles, and excitatory/inhibitory pre- and postsynaptic densities (87, 111–114). Further, to maintain active signaling complexes necessary for electrical signaling homeostasis and physiologic function, neurons rely on localized protein translation in axons/dendrites/synapses, which can be up to 1 meter in distance from the cell body (28, 115). Notably, a fraction of the intracellular RNA is associated with RBPs in condensates termed ribonucleoprotein (RNP) granules. Once these silenced RNA granules arrive at axons/dendrites/synapses, signaling-dependent PTMs regulate condensate properties, resulting in the release of RNA for either degradation or translation; this is particularly important for maintaining dendritic plasticity and regulating axon growth, regeneration, and maintenance (45, 115–117).

Tau and TDP-43 regulate biological processes within liquid-like condensates in various cellular compartments, from the nucleoplasm to the synapse (12, 76, 118–121). Interestingly, tau and TDP-43 share many functions, as revealed by an extensively similar interactome embodied by RNP complexes, RNA/protein metabolism, molecular transport, and the neuronal stress response (122–124). Tau is usually a cytosolic axonal protein, and under disease conditions, tau accumulates in postsynaptic compartments, presynaptic terminals, and the nucleus (10, 125–127). Physiologically, tau can undergo phase separation to enhance the polymerization of microtubules by condensing tubulin dimers (118, 120). This drives microtubule polymerization, after which tau dissipates onto the microtubule surface. TDP-43 exerts multiple functions, including the regulation of splicing, trafficking, and stabilization of RNA (40, 123, 128, 129). While TDP-43 typically resides in the nucleus, it also shuttles from the nucleus to the cytoplasm and is found mislocalized to the cytoplasm of diseased neurons (40, 128). Notably, TDP-43 is a component of several RNP granules, including paraspeckles, nuclear stress bodies, and RNA transport granules in neurons (12, 69, 130).

## Biomolecular condensate dysfunction

Given the essential roles that biomolecular condensates have in regulating cellular processes, one expects that many condensates are dysregulated in related diseases. Current evidence suggests that condensate dysregulation is a prevalent pathogenic mechanism underlying a broad spectrum of human diseases best described across NDDs and cancer (26, 27, 131). NDD phenotypes resulting from aging/disease-related insults include genomic DNA damage, defects in nucleocytoplasmic transport, and altered protein and RNA homeostasis (3, 16, 132–135). Under such conditions, dysregulated gene expression, alternative splicing events, disrupted RNA/protein transport, abnormal RNA/protein PTMs, and a loss in RNA/protein quality control have been observed. Many of these changes will directly impact threshold concentrations for phase separation, resulting in aberrant compositions and potential loss- and gain-of-function toxicity mechanisms. Consistent with this, pathogenic mutations across NDDs and cancer are increasingly associated with condensate dysregulation (25, 27, 136–139).

The relationship between pathogenic mutations and dysregulated condensates may be best understood by studying RBPs. Many RBPs, including TDP-43 and non-canonical RBPs like tau, are genetically linked to NDDs (22, 25, 83, 107, 140–143). Such mechanisms include enhanced driving forces for liquid-liquid phase separation and liquid-to-solid transitions, as well as altered material properties and localization of the condensates they reside within. Consistent with this notion, prolonged residency time within dense liquid-like phases was shown to increase the likelihood of liquid-to-solid phase transitions for tau, TDP-43, and other NDD-related proteins using in vitro model systems (20, 22, 23, 27, 68, 71, 108, 137, 139). However, disease-causing mutations in RBPs shift the balance of interactions between RNP assemblies, which, regardless of the mutation’s impact on liquid-to-solid phase transitions, ultimately alters condensate composition, material properties, and function (22, 23, 27, 29, 107, 144). This discovery has uncovered potentially novel mechanisms of toxicity and prompted a reexamination of loss- and gain-of-function mutations in solid-phase transitions (22, 27, 29, 38, 137, 145). Notably, the altered subcellular localization of critical condensate scaffolds can change the behavior of the scaffold and condensate components, leading to dysfunctional condensate assembly and toxicity. Additionally, disease-causing mutations may perturb the selective partitioning/exclusion of critical clients necessary for condensate assembly, localization, material properties, and subsequent function (Figure 2B).

Collectively, these discoveries have led to an exciting new framework for understanding the cellular biology underlying, and the potential molecular mechanisms driving, NDDs. Future research into dysregulated soluble protein phases containing tau/TDP-43 and other pathologic proteins will likely reveal additional links between aberrant condensates and neurotoxic mechanisms.

## Targeting aberrant phase transitions

**Figure 3:**
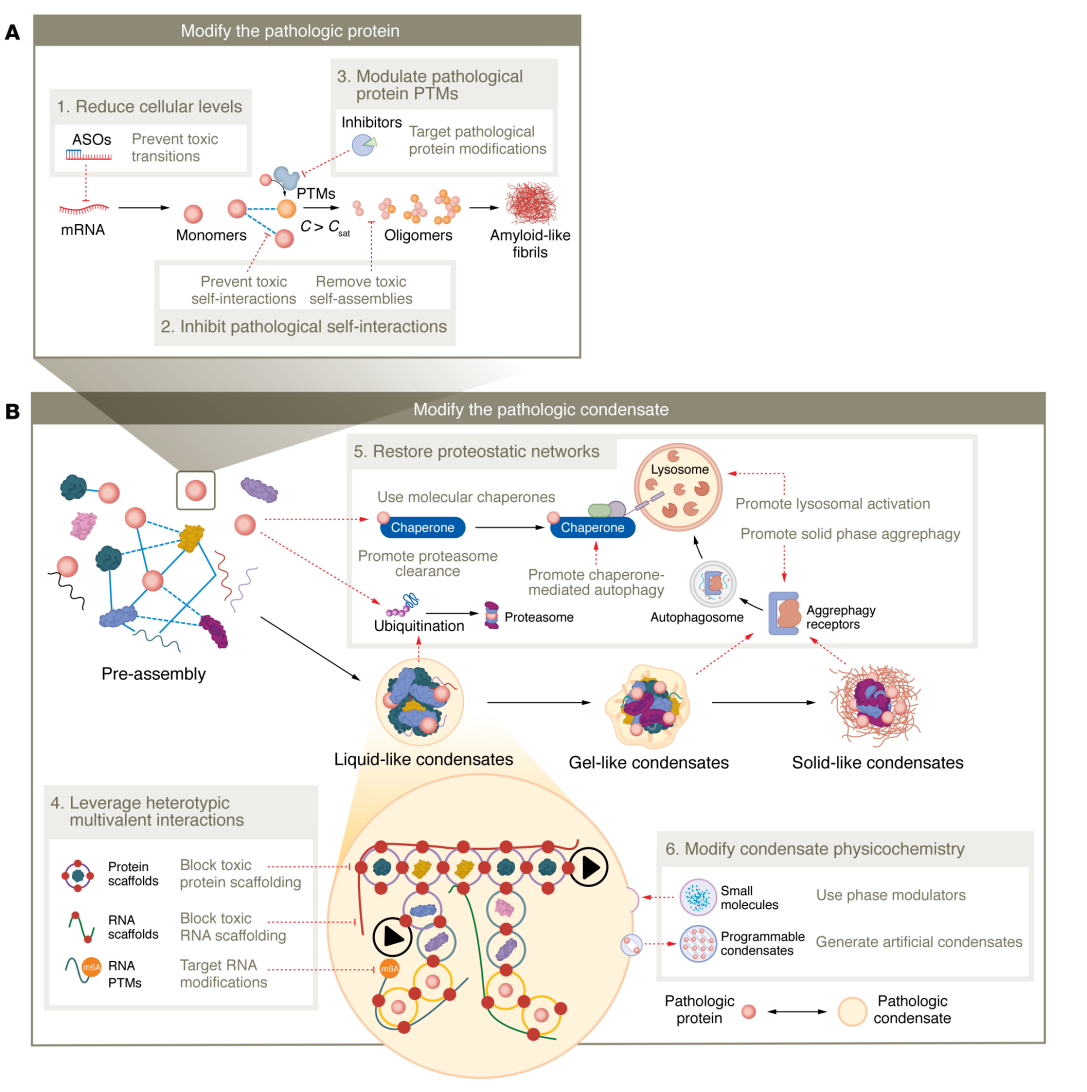
Drug discovery avenues for targeting aberrant phase transitions associated with neurodegeneration. Three major avenues for targeting pathologic protein phase transitions in NDDs are proposed. (**A**) Modify the pathologic protein. The phase behavior of a pathologic protein may be directly modified by modulation of pathologic protein levels and PTMs, and by direct targeting of toxic homotypic interactions. (**B**) Modify the pathologic condensate. With the inherent limitations of direct targeting of a single protein, modifying pathologic condensates vastly extends the pool of drug targets. The aberrant condensate features may be altered by leveraging of heterotypic multivalent interactions and physicochemical properties, and by restoration of cellular proteostatic networks.

Extensive knowledge regarding alterations to the localization and biophysical properties of tau and TDP-43 in disease has provided fundamental examples linking aberrant phase transition behaviors with toxicity and potential targets for therapeutic intervention. Review of current and potential therapeutic targeting strategies directed at tau and TDP-43 proteinopathies highlights three potential therapeutic avenues that utilize the phase transition– and condensate-based hypotheses of NDDs (Figure 3). We will first examine strategies that directly target tau and TDP-43 (Table 1). Based on the residency of these pathologic proteins within various biomolecular condensates or “pathologic condensates,” we will discuss unique strategies that leverage properties of condensate biology and the critical cellular pathways regulating biomolecular condensates (Table 2).

### Modify the pathologic protein

#### Reduce cellular accumulation of NDD-associated proteins.

As previously mentioned, protein phase transitions can be described by local saturation concentrations (*C*_sat_) and strongly influenced by protein concentration (28, 33). Therefore, it is unsurprising that both tau and TDP-43 overexpression in cellular and animal models results in neurodegeneration and is further exacerbated by disease-causing mutations (10, 146, 147). Thus, targeting RNA to reduce the cellular accumulation of NDD proteins, such as through GAPmer antisense oligonucleotides (ASOs), bypasses the many unresolved questions regarding the toxicity of specific protein conformations, modifications, and polymeric assemblies and effectively prevents downstream toxicity. For TDP-43, both motor deficits and embryonic lethality have been described after partial and complete knockdown in animal models, respectively (147, 148). Therefore, reduction of wild-type TDP-43 levels does not appear viable for clinical translation. Tau knockdown, however, has proven tolerable in many experimental models and repeatedly demonstrated cognitive protection in AD and FTD-tau_mut_ animal models (149–152). More recent work extends this protection to neuronal cultures treated with ALS synaptoneurosomes (153). Tau-lowering strategies include tau-targeting immunotherapies and RNA-targeting *MAPT* ASOs, which are currently in clinical trials for tauopathies (154). Additionally, *MAPT* isoform–specific ASOs and small molecules targeting *MAPT* RNA splicing regulatory elements have demonstrated therapeutic potential by targeting overabundant tau isoforms in rodent models of genetic forms of frontotemporal dementia (FTD) (155–157). DNA-targeting zinc finger protein transcription factors (ZFP-TFs) capable of directly targeting and lowering specific protein-coding sequences provide long-lasting reductions in tau expression following a viral-mediated introduction in disease models of tauopathy (151, 158). Embedded within most genes encoding IDPs, an endogenous mechanism exists that controls translation through the expression of natural antisense transcripts (NATs) that contain mammalian-wide interspersed repeats (MIRs) (159). These MIR-NAT sequences compete for rRNA pairing and transcript translation and may act as a potential avenue for therapeutic intervention. For example, silencing of the MIR-NAT *MAPT*-AS1 led to increased tau levels, and its expression correlated with aggregated tau in the human brain (159).

#### Inhibit pathologic self-interactions.

As discussed in previous sections, intrinsic and extrinsic factors govern the energy state of intramolecular interactions, orming physiologic protein conformations and preventing aggregation-prone conformations (5, 53, 62). Therefore, designing small molecules that stabilize physiologic protein conformations and prevent pathologic conformations, self-assembly, and subsequent deleterious phase transitions is a viable therapeutic strategy. Attempts to design IDR small-molecule modulators have proven difficult, and no clinically approved small-molecule therapeutics targeting disease-related IDPs/IDRs currently exist (160). However, studies did identify small molecules that recognize monomeric tau and TDP-43, thus supporting the possibility of this approach for future investigation (161–164). Tau monomers may occupy distinct conformational ensembles, where some conformations are relatively inert, while others have the intrinsic ability to self-assemble and are seed-competent (63, 165). The initiation of tau self-assembly likely begins with a stable transition of tau monomer from an inert to a seed-competent monomeric form. One of the most well-studied tau-interacting ligands, the small molecule methylene blue (MB) and its derivative TRx0237, has been through several phase III clinical trials (163, 166). MB and its derivatives directly interact with tau monomer, thus blocking tau-tau interactions to prevent and reverse tau aggregation in vitro (167, 168). Investigation of TRx0237 and other tau-binding small molecules highlights the potential of binding and sequestering IDPs in monomeric, soluble states. Similarly, a small molecule, nTRD22, targeting the N-terminal domain of TDP-43 was recently shown to be an allosteric modulator of TDP-43–RNA binding and conferred protection against motor deficits in an ALS-*Drosophila* model that overexpresses TDP-43 (169). These highlighted examples suggest that further research targeting monomeric forms of pathologic proteins with small molecules is a viable and promising approach to prevent and/or reverse aberrant phase transitions.

Recent work by us and others also highlights the ability of specific RNAs to regulate protein phase transitions through specific RNA-protein interactions. In the case of TDP-43, homotypic low-complexity domain (LCD) interactions initiate its pathologic aggregation through aberrant liquid-liquid phase separation, and this homotypic interaction is antagonized by RNA binding (23, 24, 76). An RNA-dependent mechanism of pathologic interaction was also shown for other NDD-associated RBPs, including FUS and tau (120, 142, 170, 171). This mechanism highlights an intriguing RNA-based targeting strategy in which an RNA aptamer or “bait oligonucleotide” might be able to engage RNA-deficient TDP-43 in the cytoplasm and prevent or reverse pathologic phase transitions. In the case of TDP-43, a bait oligonucleotide (Clip_34) comprising the *TARDBP* mRNA 3′-UTR autoregulatory domain engages the TDP-43 RNA recognition motifs and prevents neurotoxic TDP-43 self-interactions, phase transitions, and associated in vitro neurotoxicity (24, 172).

Immunotherapies to disrupt existing pathologic homotypic assemblies also showed promise for both tau and TDP-43 (161, 173–175). Tau-based immunotherapies have gone from proof-of-concept studies to clinical trials for AD and other tauopathies (154). Several notable disease-conformation-specific tau antibodies have since been developed, presenting promising results for reducing tau aggregation in preclinical models of tauopathy (176, 177). For example, the PNT001 antibody is capable of recognizing a toxic, *trans*-to-*cis* conformational change occurring early in tauopathies (178–180). PNT1001 prevents tau aggregation, neuropathology, and cognitive impairment in several preclinical tauopathy models, including models of CTE. PNT1001 is currently entering clinical trials in patients with various tauopathies, including traumatic brain injury (TBI). Similarly, a rationally developed antibody targeting an RNA recognition domain of TDP-43 was shown to successfully reduce insoluble TDP-43 inclusions, inflammation, and cognitive impairment in a transgenic ALS mouse model expressing the familial ALS TDP-43^G348C^ protein (173).

Soluble oligomeric protein assemblies of tau and TDP-43 are synaptotoxic and, in the case of tau, capable of propagating self-assembly through connected neural networks (46, 50, 54, 181–183). Recently, many rational designs leveraging stable structures mediated by LCDs/IDRs through aberrant phase transitions and the accumulation of homotypic self-assemblies have brought exciting opportunities for structure-specific targeting. Targeting the neurotoxic and misfolded protein structure and not the protein monomers should limit interference with the physiologic function of the protein when in its proper conformation. This is notable since the physiologic phase separation of IDR-containing proteins into biomolecular condensates is critical for various cellular processes. For example, physiologic phase transitions of TDP-43 into reversible biomolecular condensates is hypothesized to be essential for the binding of specific RNA sequences (23, 24, 76, 184). Thus, the development of strategies that target pathologic but not physiologic phase-separated assemblies is a powerful approach.

Regarding tau, multiple in vitro studies identified small molecules that inhibit tau assembly with various mechanisms of action. These include molecules that block inducer-specific fibril growth, preventing fibril growth by initializing nontoxic, off-pathway assemblies, and those capable of disassembling preformed fibrils (185–188). Use of cryo–electron microscopic structures of human AD tau filaments bound to small molecules has allowed the identification of novel, drug-like molecules capable of disaggregating brain-derived tau fibrils in vitro (189). One example is the small molecule Anle138b, which is currently in clinical trials for Parkinson’s disease and multiple-system atrophy and has previously been shown to reduce tau aggregation and behavioral deficits in numerous cellular and animal models of tauopathy (188). Experimental evidence demonstrated that Anle138 avoids tau monomer binding and selectively binds oligomeric tau assemblies, preventing the formation of amyloidogenic fibrils (188). Furthermore, crystal structures of tau steric zippers led to the rational design of small steric zipper–binding peptides, referred to as “fibril capping” peptides (190).

#### Modulate pathologic protein PTMs.

PTMs, including covalent modifications and cleavage events, offer a fine-tuned response to diverse extracellular stimuli and intracellular signaling pathways (45, 191–193). PTMs substantially alter the intrinsic properties of a sequence and thus regulate intra- and intermolecular interactions (62). Therefore, covalent modifications can act as potent regulators of protein/RNA conformations and, consequently, the properties of biomolecular condensates. In disease, tau and TDP-43 are often found heavily modified by PTMs (phosphorylation, acetylation, ubiquitination, etc.) and cleaved into fragments (10, 53, 193–197). While the effect of PTMs on biomolecular phase behavior is only beginning to be understood, PTMs may directly regulate phase behavior by altering either intra- or intermolecular interactions, leading to an altered *C*_sat_. Lysine-modifying acetylation in tau and TDP-43 significantly reduces critical lysine-RNA interactions, resulting in altered phase behaviors (198–200). Targeting of tau acetylation after TBI using acetylation-inhibiting drugs (salsalate) is associated with reduced neurodegeneration in humans and prevents tau mislocalization, insolubility, and cognitive deficits in preclinical models (201). TDP-43 acetylation, which mitigates RNA binding, enhances its phase separation into complex nuclear droplets called anisomes that colocalize with HSP70 and can promote aberrant phase transitions when localized to the cytoplasm. This results in gel-like and insoluble assemblies and highlights the role of RNA binding as a modulator of TDP-43 liquid-liquid phase separation (24, 200, 202). Importantly, pairings of PTMs may have distinct effects on downstream modifications, either stimulating or inhibiting hallmark phase transitions (53, 197, 203, 204). PTM-modifying therapies will require extensive study with regard to the complex interplay between single and combinatorial PTMs and how PTMs alter biomolecular interacting partners, resulting phase behaviors, and subsequent neurotoxicity.

### Modify pathologic condensates

#### Leverage heterotypic multivalent interactions.

The growing knowledge regarding condensate assembly and regulation opens avenues for interfering with pathologic phase transitions. With the inherent limitations of direct targeting of pathogenic phase transitions of a single protein (tau or TDP-43), targeting biomolecular condensates that might drive aberrant phase transitions vastly extends the pool of drug targets. The residency and scaffolding potential of pathologic proteins in critical cellular condensates are intriguing. Modifying condensate scaffolds would significantly affect condensates’ stability, including assembly, dissolution, material properties, and composition of scaffold/ligands/etc. (28, 43, 73, 79, 205). Thus, disrupting specific components and regulatory pathways of biomolecular condensates to indirectly modify abnormal hallmark phase transitions and cellular toxicity may be a therapeutic approach. The ultimate goal of this is to shift tau or TDP-43 *C*_sat_ and phase transition behaviors.

Scaffold modulation can be achieved in several ways. Approaches may include preventing or stabilizing protein-protein, protein-RNA, and RNA-RNA interactions that contribute to condensate scaffolding. Intriguingly, the genetic manipulation of RBPs often alters the rate of tau and TDP-43 aggregation in several model systems (206–209). TIA1 is an RBP and a major component of stress granules (SGs). Previous studies indicate that TIA1 interacts with tau, and this interaction modulates tau aggregation and toxicity (208, 210, 211). TIA1 knockdown prevents tau-mediated toxicity, reduces toxic soluble tau oligomers, and increases insoluble tau fibrils (208). Importantly, tau fibrils isolated from the diseased brain contain numerous RNA species. Recent research has demonstrated tau-mediated disruptions in RNA metabolism, leading to tau-RNA accumulations building on the nuclear envelope (212, 213). Remarkably, promoting nonsense-mediated mRNA decay with a small molecule, tranilast, disrupts these tau-RNA accumulations, suppressing neurodegeneration and locomotor deficits in a tau-transgenic *Drosophila* model.

Ataxin-2 (ATXN2) is an RBP found in mature SGs, and intermediate CAG expansions within the *ATXN2* gene are found in subsets of ALS cases (214). Recent work found that ATXN2 knockdown reduces abnormal SG formation and is neuroprotective in both in vitro and in vivo rodent models with elevated levels of TDP-43 (209). Additionally, ATXN2 reduction significantly reduces TDP-43 pathology. Further, ALS’s most common genetic cause (expansions of C9orf72) leads to the overexpression of expanded GC RNA repeats, leading to TDP-43 mislocalization, assembly, and toxicity (215). Recently, a small-molecule-guided ribonuclease-targeting chimera (RIBOTAC) method capable of directly targeting the removal of G_4_C_2_ duplications prevented TDP-43 insolubility and neurotoxicity in animal models (216). Additionally, recent work has demonstrated that upregulating an endogenous TDP-43–interacting noncoding RNA, NEAT1_1, lowered TDP-43 insolubility and toxicity in *Drosophila* and yeast models of TDP-43 proteinopathy (217). Together, this suggests that pathogenic interactions within biomolecular condensates may promote aberrant TDP-43 and tau phase transitions and that modulating these interactions might confer neuroprotection and be a potential therapeutic approach.

While considerable attention has been focused on protein modifications, recent work highlights a long list of covalent nucleic acid modifications that may alter TDP-43 and tau phase transitions (78, 218–222). DNA and RNA methylation are potent regulators of nucleic acid phase separation and affect the condensation properties of specific protein–nucleic acid complexes (221, 223, 224). Interestingly, the knockdown of the canonical RNA *N*^6^-methyladenosine (m6A) reader YTHDF2 was recently shown to prolong the survival of induced pluripotent stem cell human neurons carrying ALS-associated mutations (225). Consistent with this, knockdown of the canonical RNA m6A reader HNRNPA2B1 and the m6A writer METTL3 rescued tau-oligomer-induced neurodegeneration in models of tauopathy (206). Thus, the targeting of these RNA modifications is slowly being revealed as a novel approach capable of regulating pathologic protein phase transitions.

#### Restore proteostatic networks.

The proteostasis network, a protein quality control (PQC) system, regulates and balances protein synthesis, folding, transport, and degradation (226–228). Impairment of one or several PQC mechanisms can result in aberrant phase transitions and the accumulation of protein aggregates inside neurons. The PQC system is an integrated network of molecular chaperones, co-chaperones, and two degradative systems, the ubiquitin-proteasome system (UPS) and autophagy, a lysosome-mediated bulk degradation pathway (226, 229). Traditionally, autophagy was believed to preferentially clear protein aggregates with a certain amount of “liquidity” in a process referred to as aggrephagy (230–232). An arm of aggrephagy was recently discovered and selectively targets protein aggregates with little liquidity (solids) for lysosomal degradation, thus highlighting critical cellular mechanisms that interact with biomolecular condensates with specific intrinsic material properties (231). While aggrephagy was thought to process condensates with some liquidity, recent work demonstrated that the CCT2 autophagy receptor allows for the selective targeting of solid condensates. Notably, the upregulation of CCT2 cleared several solid protein aggregates from cells, including mutant tau protein (231).

Several pharmacologic agents that modulate the ATPase activity of HSP70, a core chaperone, have been designed and tested in NDD models (233, 234). Interestingly, reduction of HSP70 ATPase activity transforms TDP-43 liquid phases into gel-like structures, leading to insoluble TDP-43 assemblies and increased toxicity (200). Substantial efforts found that non-core chaperones, including a specific class, the peptidyl-prolyl *cis*-*trans* isomerases (PPIases), protected against aberrant tau phase transitions (235). Specifically, Pin1 catalyzes proline *cis*-to-*trans* isomerization, a conformational change that protects against the stabilization of toxic conformations that lead to pathologic tau fibrils (176, 177, 179). Increasing evidence shows that nuclear-import receptors chaperone and disaggregate RBPs, including TDP-43 (40, 236, 237). Not only do nuclear localization sequences (NLSs) mediate the nuclear import of NLS-containing proteins, but they also inhibit deleterious phase transitions and promote the disaggregation of solid assemblies. In the cytoplasm, specific nuclear-import receptors that engage the TDP-43 NLS, importin-α and -β, prevent and reverse TDP-43 aggregation n models of C9orf72 ALS/FTLD (236).

The UPS predominantly regulates soluble tau and TDP-43, and the accumulation of these species can lead to protein nucleation (238–242). While macroautophagy pathways can directly sequester and degrade larger condensates, soluble protein monomers can be degraded by chaperone-mediated autophagy (CMA). The inability to remove accumulating soluble proteins eventually promotes the aggregation of the CMA-regulated proteome. Consistent with this, CMA deficiency in the aging brain is an aggravating factor in the onset of NDD (243). Activation of CMA with small molecules has proven neuroprotective in animal models of tauopathy (243, 244).

#### Modulate condensate physicochemistry.

While the direct engagement of condensate components may allow a prospective drug to occupy a condensate, a drug may also concentrate within a condensate due to a network of transient contacts without high affinity toward a specific target (101, 245–249). Therefore, a small molecule, through interactions with the chemical environment of the condensate, may strongly influence condensate properties regulating the formation or dissolution of condensates. Therefore, using small-molecule ligands to target condensates may be a promising therapeutic strategy. This premise is clearly illustrated by cellular metabolites like ATP, cAMP, glucose, and many others, which were previously demonstrated to modulate condensate properties (34, 43, 73, 250–252). Several known small molecules can alter the phase behaviors of tau and TDP-43 proteins by either directly interfering with the ability of the pathologic protein to self-condense into liquid-like phase, or interfering with their recruitment to biomolecular condensates (i.e., SGs) (245–248). Specifically, molecules with planar moieties, such as mitoxantrone, were shown to prevent TDP-43 cytoplasmic localization and prolonged residency in SGs (246). Further, the compound myricetin can slow the liquid-like phase separation of tau, shifting its phase boundary while stabilizing the interaction of tau protein within the aggrephagy clearance pathway (249). Besides regulating the properties of existing hallmark condensates with small molecules, interest in generating artificial condensate systems to engage with endogenous condensates is growing. Interestingly, the cytoplasmic expression of the neuronal chaperone proSAAS created micron-scale membraneless spheres with condensate features that selectively encapsulated and sequestrated TDP-43 aggregates and reduced their toxicity in cell culture models (253). Further work is under way designing programmable condensates capable of sequestering pathologic aggregates, stabilizing the pathologic proteins’ normal physiology, and facilitating drug delivery and enrichment toward specific condensates.

## Conclusion

It is believed that the biochemical changes responsible for initiating NDDs begin decades before the clinical presentation (2, 9, 26, 226, 228, 254). Furthermore, there are fundamental challenges to differentiating “normal” age-related events from pathologic biochemical processes that drive the earliest stages of neurodegeneration or distinguishing primary causes from a cascade of secondary insults. Aberrant protein conformations, oligomers, and fibrils composed of neuropathologic protein depositions may symbolize both a symptom and a cause of the underlying disease. As new discoveries emerge describing structure-specific protein assemblies in NDD subtypes, a thorough understanding of the cellular conditions driving these unique self-assemblies will prove important to develop disease-modifying therapies (56, 57, 64, 255). Condensate biology is fundamental to numerous cellular processes, and a growing understanding of these mechanisms is already transforming our understanding of how cells spatiotemporally organize biomolecules to regulate critical cell functions. As the formation of biomolecular condensates involves and influences all levels of macromolecular organization, condensate biology can profoundly expand our understanding of the pathologic conditions that lead to toxic protein assemblies, resulting downstream cellular dysfunction, and subsequent neurodegeneration. Targeting of aberrant phase transitions as a therapeutic intervention for neurodegenerative disorders will require substantial work to better characterize the diverse condensate subtypes and their components, physicochemical properties, assembly mechanisms, and physiologic function.

## Author contributions

BTH and LX conceptualized and outlined the contents of this review, figures, and tables. BTH wrote the initial full draft with input from LX. BTH and LX both addressed reviewers’ comments.

## Figures and Tables

**Table 2:**
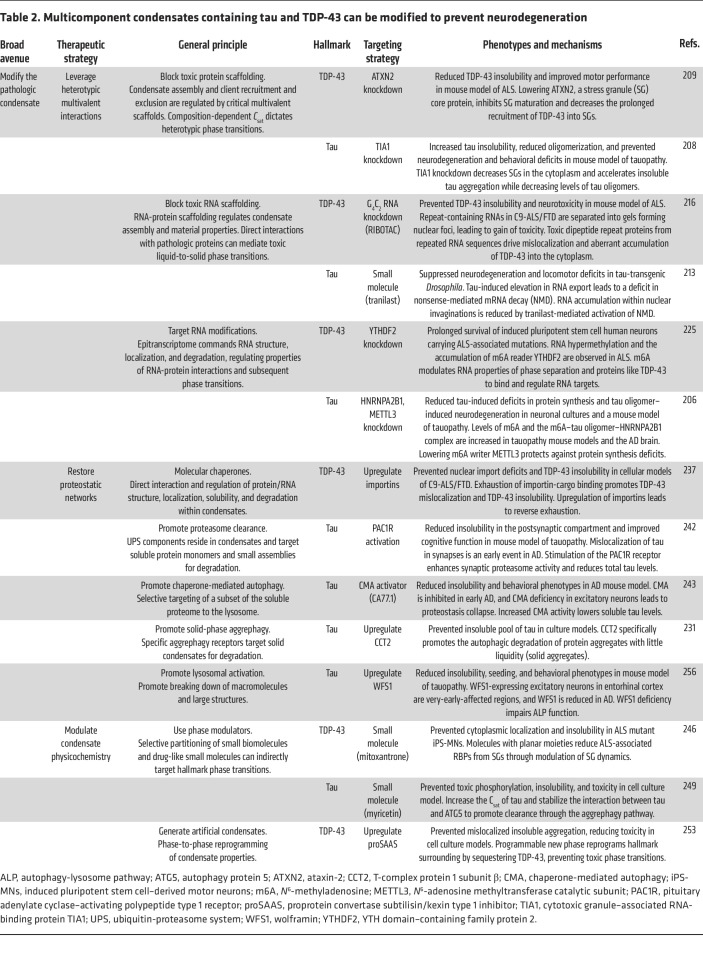
Multicomponent condensates containing tau and TDP-43 can be modified to prevent neurodegeneration

**Table 1:**
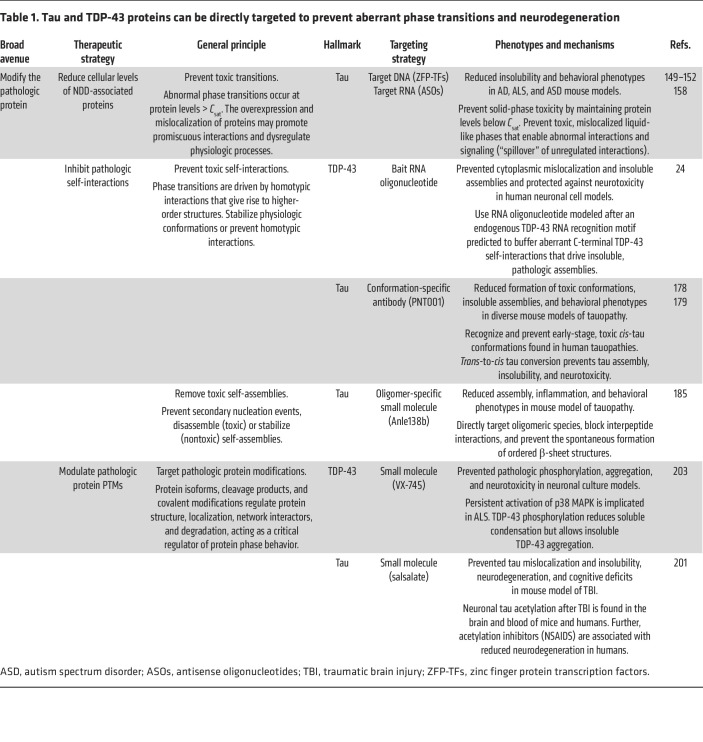
Tau and TDP-43 proteins can be directly targeted to prevent aberrant phase transitions and neurodegeneration

## Version 1 07/03/2023

Electronic publication
